# Oviposition activity of *Drosophila suzukii* as mediated by ambient and fruit temperature

**DOI:** 10.1371/journal.pone.0187682

**Published:** 2017-11-09

**Authors:** Florian N. Zerulla, Clemens Augel, Claus P. W. Zebitz

**Affiliations:** Department of Applied Entomology, University of Hohenheim, Stuttgart, Baden-Württemberg, Germany; Institut Sophia Agrobiotech, FRANCE

## Abstract

The invasive pest *Drosophila suzukii* was introduced to southern Europe in 2008 and spread throughout Central Europe in the following years. Precise reliable data on the temperature-dependent behavior of *D*. *suzukii* are scarce but will help forecasting and cultivation techniques. Depending on physico-chemical properties, surface temperature of objects may differ from ambient temperatures, determining physical activity, and affect oviposition on or into substrate, determining preimaginal development later. Therefore, the preferred ambient temperatures of *D*. *suzukii* and fruit temperature for oviposition were examined on a linear temperature gradient device. Thirty adults (15 ♀; 15 ♂) were adapted to different temperatures (10, 20, 30°C) for six days and then exposed to different temperature gradients (10–25, 20–35, 25–40°C). *D*. *suzukii* adapted to 10°C remained in cooler regions and suffered from a significantly higher mortality at the 25–40°C gradient. Animals adapted to warmer temperatures had a wider temperature preference on the gradient device. Acclimation to lower temperatures and the resulting lower temperature preferences may allow the flies to disperse better in spring to search for oviposition sites. The oviposition activity decreased continuously at a fruit temperature above 28°C and below 15°C, with highest oviposition activity in fruits with temperatures between 19.7°C and 24.8°C. The preferred fruit temperature is in accordance with the temperature optimum of reproduction biology and preimaginal development of *D*. *suzukii* reported in the literature.

## Introduction

One of the most serious pests of grape, berry and stone-fruits in recent years is *Drosophila suzukii* (Matsumura) (Diptera: Drosophilidae), which originates from Southeast Asia. This species has been introduced into several countries of the Old and New World and has become an invasive species [1].

Damage caused by *D*. *suzukii* in Europe was first reported in 2008 from Spain and Northern Italy [1, 2, 3]. The first individuals may have arrived in infested fruits as eggs or larvae which had been sea-traded from Asia [4]. Since the first recordings, *D. suzukii* has now spread over North and South America, Asia and Europe [1, 5, 6].

Most *Drosophila* species, like *D*. *melanogaster*, oviposit on rotten, overripe and damaged fruits. In contrast, *D*. *suzukii* females possess a sharp and serrated ovipositor, which allows them to pierce the skin of intact fruit and lay eggs into fresh fruits before harvesting and thereby damage the fruit skin actively [7]. Feeding damage by the larvae as well as by secondary infestations by other insect pests (e.g. wasps) and pathogens can lead to a yield loss of up to 100% [8, 9]. Due to the high reproduction rate of *D*. *suzukii* of up to 500 eggs/female, large economic losses may occur [1, 10, 11]. Insecticide applications can prevent damage but drive up production costs [3, 8, 9, 12, 13]. Restrictions in application frequency during fruiting and pre-harvest interval, short persistence, or residue levels rising the risk of rejection of fruits in the market, do not allow effective long-term control [14, 15]. Combinations of pest management measures may increase control efficacy but are considered economically or ecologically prohibitive [13].

Under optimum environmental conditions the life-cycle is completed in about 9 to 11 days. This short generation time leads up to 13 generations per season [16]. Additionally, the large number of wild and cultivated host plants like blueberries, elderberries, raspberries, blackberries, grapes and figs render this pest difficult to control [3, 17, 18, 19]. Yet there is no effective option to prevent infestation of crops at risk or nearby wild host plants by *D*. *suzukii*. The insufficient knowledge of the biology of *D*. *suzukii* under European environmental conditions might explain the lack of effective control. It is known that the ambient temperature has a significant impact on development time, survival and fecundity, however, wide gaps in knowledge exist on the adaptability of *D*. *suzukii* under low and high temperatures. Speed of development of *D*. *suzukii* is strongly temperature dependent [9]. Temperature optima for preimaginal development, mortality and fecundity have been found, ranging from ca. 25 to 28°C [10, 20]. Temperatures above ca. 30°C were reported to have detrimental effects on reproduction, preimaginal development, and survival. In addition to speed of development, mortality and fecundity, temperature also controls physical activity such as locomotion, foraging, mating and feeding propensity, where thresholds and optima have not been clearly defined for *D*. *suzukii* yet [10, 21, 22].

In the field, *D*. *suzukii* are exposed to rapid temperature changes at different geographic regions and time, such as living at various altitudes, vast differences between day and night, or by season. It may be assumed, that *D*. *suzukii* is not fixed to a particular specific temperature, but prefers a temperature range, which has not been defined yet, except for preimaginal development and mortality [20].

The goal of the studies shown here was to understand temperature preference of *D*. *suzukii*. Adult flies were given a choice of different temperatures after previous adaptation to different ambient conditions to examine whether temperature experience can modify preferences.

A second experiment was set up to clarify whether the fruit temperature mediates the choice of oviposition site by *D*. *suzukii*. Fruit surface temperature often differs from that of the flesh and ambient temperature [23]. Although temperature sensillae near or on the ovipositor have not been reported for Diptera yet, we hypothesize detection of surface temperature to be the determining factor rather than the temperature inside the fruit. Consequently, the test set-up included measuring the preference for an oviposition site by counting should be seen in the number of eggs on blueberries of different fruit-temperatures.

Changes in temperature, particularly during spring, affect oviposition activity and, thus, population dynamics, which could increase or decrease the damaging potential of *D*. *suzukii* during summer and autumn when effective control is more difficult.

## Material and methods

### *Drosophila suzukii* rearing

*Drosophila suzukii* was reared in the Institute of Phytomedicine, University of Hohenheim, Germany since 2014. Annually, summer and fall field-trapped individuals were used to supplement the lab-rearing to avoid genetic bottlenecks. The flies were reared in gauze cages (50 x 40 x 50 cm, width x depth x height) in a climatic chamber at constant 20°C and 80 ± 5% RH at a 16:8 h light-dark regimen. A piece of artificial yeast diet (ca. 5 × 2 × 3 cm, width × depth × height) (modified *Cydia pomonella* diet after Bathon et al. [24]) was offered for 2–3 days as oviposition substrate. A cotton-pad soaked with 6% sucrose-water solution served as carbohydrate/water source. Yeast diet pieces with eggs were placed in a plastic box and kept under rearing conditions until pupation. After adult emergence, flies were released into fresh cages.

### Temperature preference experiments

To assess temperature preference, the adult *D*. *suzukii* were given a choice on a temperature gradient bar, which offered the opportunity to set up different temperature ranges.

#### Temperature gradient bar

**Fig 1:**
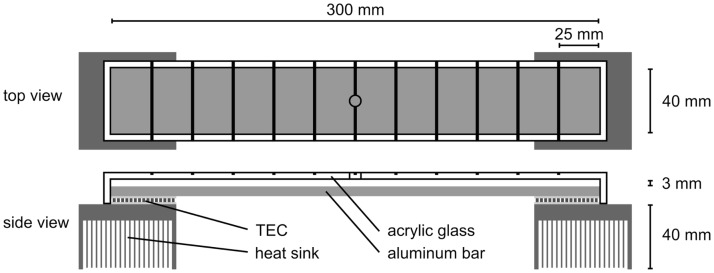
Temperature gradient bar, divided into 12 sectors.

The temperature gradient bar was made of a sandblasted and matt-black painted aluminum sheet (300 x 40 x 5 mm length x width x thickness) with two thermoelectric coolers (TEC1-12706; 40 x 40 x 3.8 mm) attached to each end. Heating or cooling the TECs to different temperatures by a laboratory power supply (LT30-2; 2x 30V 2A; Farnell Instruments Ltd.) resulted in a linear temperature gradient along the aluminum bar (Fig 1). Waste heat produced at the underside of the gradient-cooling TEC was dissipated by a fan-cooled heat sink. The gradient bar was divided into 12 sections (25 x 40 mm each) by fine pencil markings to facilitate temperature recording of the current residential position of test individuals. This was particularly necessary for flies still moving or aggregating, and for the experiments on oviposition, where the fruits alone claimed more area than a single adult fly. Furthermore, the temperature-difference (mean ± s.e.m.) between neighboring sections in the temperature preference experiments was 1.34 ± 0.03, 1.38 ± 0.02, and 1.46 ± 0.02°C for the gradients 10–25, 20–35, and 25–40°C, respectively. The temperature-difference (mean ± s.e.m.) between neighboring sections in the oviposition preference experiments was 1.02 ± 0.06 and 1.29 ± 0.06°C for the gradients 12–26 and 18–36, °C, respectively. These differences were acute enough and less than the overall variance of temperature preferences within a temperature gradient. Temperature in all experiments was assessed with a non-contact digital infrared thermometer (Lasergrip 774, Etekcity, Anaheim, USA).

#### Ambient temperature preference by conditioned adults

For 6 ± 1 days, 2 ± 1 days old adult male and female *D*. *suzukii*, taken randomly from a synchronized stock laboratory culture, were adapted to temperatures of either 10, 20 or 30°C in a climatic chamber (80 ± 5% RH at a 16:8 h light-dark regimen). During this time, the flies were supplied with yeast diet and a sugar-water solution *ad libitum*. The temperature gradient bar was placed in an air-conditioned room at 22.5°C and was brought to temperature ranges of 10–25°C, 20–35°C, and 25–40°C. An acrylic glass roof was placed 3 mm above the aluminum bar to prevent flies from escaping. Fifteen ♀ and ♂ adults each, taken randomly from the temperature-adapted synchronized stock laboratory culture, were then transferred into the space left through an opening in the center of the roof. The initial distribution of flies, regardless of their sex, in each section was assessed one minute later and the dispersal to preferred temperatures in 5 minute intervals until the end of observation after 60 min. Five replicates, each of 15 male and female flies, were conducted per each temperature gradient (10–25, 20–35, and 25–40°C) and for each adaptation temperature (10, 20, and 30°C).

#### Oviposition-site selection

To assess temperature preferences for oviposition, 2 ± 1 days old adult *D*. *suzukii*, taken randomly from a synchronized stock laboratory culture and supplied with sugar water and artificial diet *ad libitum*, were allowed to mate for five days under rearing conditions. In this experiment, the temperature gradient bar was placed in a climatic chamber at 20°C, 80 ± 5% RH. For each replicate 12 randomly chosen blueberries were prepared by cutting off a small slice at the fruit stalk in order to increase the contact area between the fruits and the temperature bar and one blueberry each was placed in the sections of the gradient bar. After reaching a fruit surface temperature in a range of 12–26°C and 18–36°C, an acrylic glass casing was put over the gradient bar, creating a 600 cm³ space (300 x 40 x 50 mm). Subsequently, 25 mated females were released into this space and allowed to oviposit on the blueberries for 21 h (11 h photophase, 8 h scotophase, 2 h photophase). Then the fruits were removed from the temperature gradient bar and the number of eggs laid into each fruit was determined under a binocular microscope by counting the filamentous spiracles reaching the fruit surface [9]. Experiments were repeated eight and nine times for the temperature ranges of 18–36 and 12–26°C, respectively.

### Statistical analyses

All obtained data were analysed using JMP^®^ 11.0.1 software (SAS Institute Inc., Cary, NC, USA). Before statistical analysis the residuals were tested for normal distribution by Shapiro-Wilk test. All continuous data, including percentage values, were found normally distributed.

The percentage of fly mortality in the above studies was also tested for the effects of temperature range and adaptation temperature by Kruskal-Wallis non-parametric oneway analysis of variance followed by Wilcoxon’s each pair test at α = 0.05.

To determine temperature preferences, the percentage of adults in each temperature section was calculated per replicate (no. of adults in one section/total adults). A bivariate regression tested the relationship between the percentage of adults in a given section and its temperature. If necessary, X or Y data were transformed to find the best fitted line. Best fit of the model was proved by “Lack of Fit” sum of squares (difference between “Total Error” and “Pure Error”) divided by degrees of freedom for “Lack of Fit” (d.f. difference between “Total Error” and “Pure Error”). The ratio of mean square for “Lack of Fit” to mean square for “Pure Error” provides the F-value. A high *p* value (P > F-value) indicates no significant lack of fit of the model (JMP-Procedure “Lack of Fit Report”). Nine regressions were tested, one for each gradient and adaptation combination.

The same procedure was followed in the oviposition experiments.

Residuals of continuous data were found normally distributed. All data were subjected to an analysis of variance, procedure “Generalized Linear Models”, before ensuing statistical analyses. The respective statistical procedures and statistical core data are provided in the legends of the tables.

#### Models

Since the proportion of eggs from the total number of eggs deposited plotted against the respective fruit surface temperature of both gradients suggested a normal distribution, the data were subjected to a nonlinear regression using an iterative Gauss-Newton model

$$\%\;oviposition=h\times \text{exp}\left[\bar{\frac{-(temperature\;{}^{\circ}C-x0)²}{\left(2\times w²\right)}}\right]$$ (1)

with h = height of the curve’s peak (= maximum % oviposition), x0 = position of the centre of the peak (temperature with highest % oviposition), and w = standard deviation.

The normal distribution of oviposition rates allows the calculation of cumulative oviposition rates over both temperature gradients and a subsequent subjection of these data to a probit-analysis. The resulting equation permits the calculation of temperature ranges for oviposition activity, where the respective probits can be taken for any percentage of cumulative oviposition to calculate the corresponding temperature.

## Results

### Temperature preference

Fly mortality of conditioned adults in the temperature preference experiments was significantly affected by temperature range (F = 19.8; d.f. = 2, 39; p < 0.0001), adaptation temperature (F = 21.9; d.f. = 1, 39; p < 0.0001), and interaction of both (F = 19.9; d.f. = 2, 39; p < 0.0001). The highest mortality of 7.33% was found in the experiments with 10°C-adapted flies followed by 1.56% mortality for 20°C-adapted flies. The lowest mortality of 0.44% was found in the experiments with 30°C-adapted flies (Table 1).

**Table 1:** Fly mortality (%, mean ± s.e.m.) of conditioned adults in the temperature preference experiments. (Kruskal-Wallis oneway analysis of variance for mortality per treatment: Chi^2^ = 27.624, p < 0.0006, d.f. = 8; all followed by Wilcoxon each pair test, α = 0.05. n = 5 in all experiments. Different letters indicate statistically significant differences in a line.)

adaptation temperature	10°C			20°C			30°C		
**temperature gradient (°C)**	10–25	20–35	25–40	10–25	20–35	25–40	10–25	20–35	25–40
**mortality (treatment) ± SEM**	0.7 ± 0.7 bc	0.7 ± 0.7 bc	20.7 ± 4.4 a	0.7 ± 0.7 bc	0 c	4 ± 1.6 b	0.7 ± 0.7 bc	0 c	0.7 ± 0.7 bc

**Fig 2:**
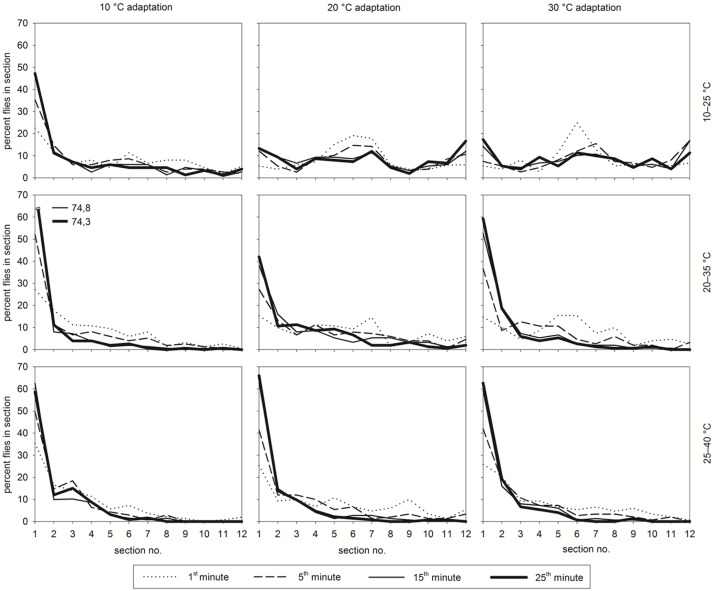
Distribution of *D*. *suzukii* (%) after adaptation to 10, 20, or 30°C across temperature sectors of three temperature gradients (10–25, 20–35, and 25–40°C).

During the experiments after *ca*. 25 min, almost all live *D*. *suzukii* have chosen their final preferred temperature and did not change their position thereafter. Thus, the following graphs show the time of data acquisition between 1 and 25 minutes (Fig 2). In the temperature gradients adults aggregated near the adaptation temperature. Whenever temperatures on the gradient bar at the cooler end were near the adaptation temperatures, the final preferred temperature was found within sections 1–5 and was significantly negatively correlated with temperature (Table 2). The distribution of adults in sections 6–12 did not change significantly and the slope of correlation equations were near 0 (not given in a table).

**Table 2:** Preference (%)–temperature (°C) correlations for sections 1–5 of the gradient bar of *D*. *suzukii* adults adapted to different temperatures exposed to three different temperature gradients.

Gradient (°C)	Adaptation temperature (°C)	F	d.f.	p	Equation	r^2^
**10–25**	10	42.891	1, 19	< 0.0001	log(%) = 13.72 − 4.35 × log(°*C*)	0.693
	20	2.868	1, 21	0.105		
	30	3.568	1, 23	0.072		
**20–35**	10	53.973	1, 20	< 0.0001	log(%) = 44.66 − 13.74 × log(°*C*)	0.730
	20	13.392	1, 20	0.002	log(%) = 18.40 − 5.09 × log(°*C*)	0.401
	30	44.034	1, 23	< 0.0001	log(%) = 33.95 − 10.23 × log(°*C*)	0.657
**25–40**	10	16.947	1, 21	0.0005	log(%) = 31.41 − 8.73 × log(°*C*)	0.447
	20	40.090	1, 18	< 0.0001	log(%) = 48.62 − 14.04 × log(°*C*)	0.690
	30	31.641	1, 20	< 0.0001	log(%) = 51.02 − 14.77 × log(°*C*)	0.613

#### 10°C—Adaptation

25 min after release, temperature preference of adults adapted for 6 days to 10°C after emerging was clearly negatively correlated with temperature of the test arena ranging between 10–25°C (Table 2). 47.2% preferred a temperature of 10°C, whereas only 25.3% were found in temperatures between 11–15.4°C (sector 2–5) (Fig 2).

On the temperature bar with a heat gradient between 20–35°C, 1 min after release into the arena, 73.3% of the adults had chosen the coolest sector. However, within the following 15 min, the adults dispersed and chose a wider range of the cooler sectors. The warm sectors were totally avoided (Fig 2).

An almost similar result was obtained when adults were exposed to a temperature range between 25–40°C (Fig 2). When entering the hot sectors near 40°C, adults often could not escape from heat and died from heat shock (Table 1).

#### 20°C—Adaptation

In the temperature range of 10 to 25°C, in the first minute more than 50% of *D*. *suzukii* adapted to 20°C remained in the sections of a temperature range of 16.5–19.3°C (sector 5–7). Since this gradient is near the adaptation temperature the adults showed an even temperature preference over the whole gradient. This is expressed by a slope near zero in the linear preference—temperature regression curve over the whole gradient (%*adults* = 9.32 − 0.05 × °*C*%; r^2^ = 0.0014; n = 60).

After offering *D*. *suzukii* the temperature range of 20–35°C, in the first minute an average of 19 flies (= 66.3%) were found in the cool section 1 followed by the warmer section 7 (27.8°C) (15.3% and 14.7%, respectively) (Fig 2).

A similar trend of temperature preference found for the temperature range of 20–35°C was observed for the temperature range of 25–40°C (Fig 2).

#### 30°C—Adaptation

*D*. *suzukii* adapted to 30°C mainly preferred a moderate temperature between 17.9 and 19.3°C (sector 6–7) on the 10–25°C gradient shortly after release.

Until 25 min after release, the adults showed an even temperature preference over the whole gradient with no distinct temperature preference, which is expressed by a slope near zero in the linear preference—temperature correlation over the whole gradient (%*adults* = 11.52 − 0.18 × °*C*; r^2^ = 0.014; n = 60) (Fig 2).

One minute after release into the 20–35°C gradient, *ca*. two third of adult *D*. *suzukii* have chosen the cooler part of the gradient and one third was found on the warmer part. In the course of the first 15 minutes the number of individuals strongly rose in the cooler sections, particularly in the first section, resulting in 17.8 flies (= 59.3%) in the coolest temperature 25 min after release, reaching a maximum of 19.8 flies (= 66.0%) at the end of the observation period.

One minute after starting the observation in the warmest temperature gradient (25–40°C), almost all *D*. *suzukii* were found in the cooler sections. The number of individuals in section 1 increased within the first 20 minutes from 26 to 64.7%, and nearly all *D*. *suzukii* were found in the first five cooler sections.

Conspicuous behavioral differences were observed on the warmest temperature gradient of 25–40°C. In sections with temperatures above 35°C, *D*. *suzukii* adapted at 10°C, began to jump around and tried to escape from these high temperatures. These flies then often landed on their dorsal side and could not turn to their feet by flapping their wings. Most of these individuals died after a few seconds.

In contrast to individuals adapted to 10°C, *D*. *suzukii* adapted to 20°C stayed for a short time only in the sections with temperatures above 35°C and then moved into sections with lower temperatures.

Flies adapted to 30°C tolerated temperatures above 35°C apparently without problems. Generally, it was observed that flies died in sections with temperatures above 40°C after a few seconds. Particularly between the two temperature gradients of 10–25°C and 25–40°C it could be observed, however it was not quantified, that walking speed of flies clearly increased in the warmer gradient. It was also observed that mating occurred occasionally at temperatures of 20 to 27°C.

### Oviposition at different fruit temperatures

**Fig 3:**
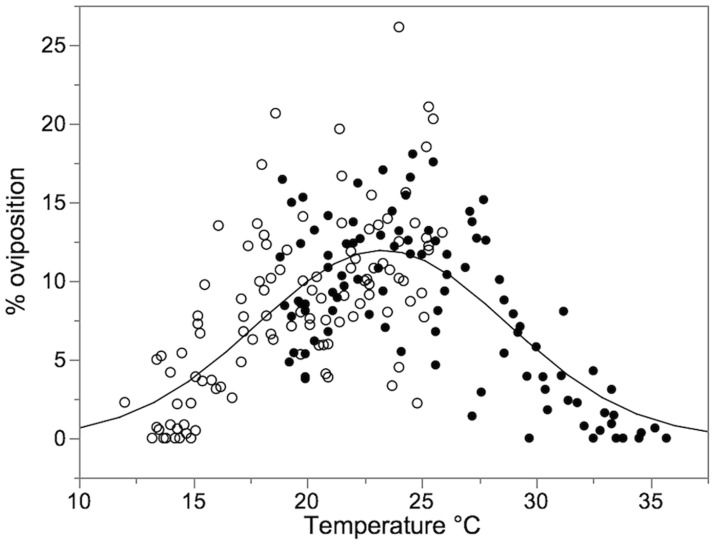
Temperature-dependent oviposition rates of *D*. *suzukii*-females on a 12–26°C gradient (open symbols) and on a 18–36°C gradient (closed symbols). The line represents the calculated Gaussian distribution (h × exp[(−temperature − x0)^2^ ÷ (2 × w^2^)]; for h = 11.937; x_0_ = 23.28; w = 5.486; N = 204; RMSE = 3.798; alpha = 0.05; convergence criterion: 0.00001).

Percent oviposition in both, the cooler and the warmer temperature gradient followed a Gaussian distribution when plotted against section temperature (11.94 × exp[(−*temperature* − 23.28)^2^ ÷ (2 × 5.49^2^)]; N = 204; RMSE = 3.798; alpha = 0.05; convergence criterion: 0.00001). The temperature-dependent oviposition rates of *D*. *suzukii*-females on a 12–26°C gradient and on a 18–36°C gradient are presented in Fig 3.

**Fig 4:**
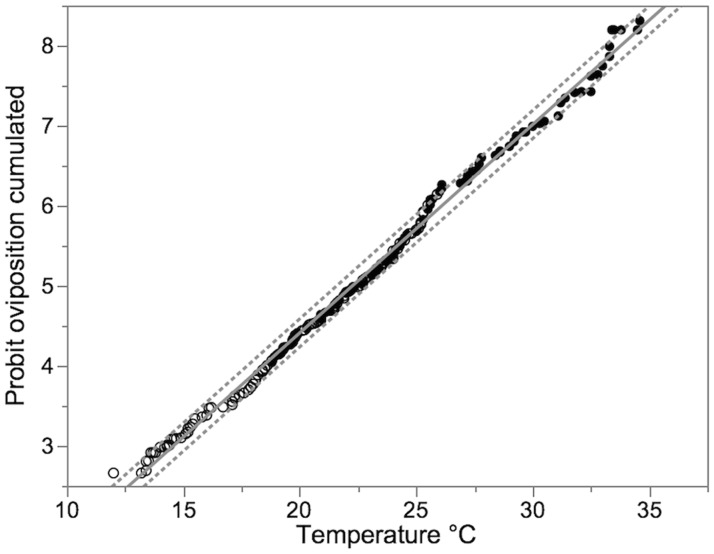
Linear correlation of cumulated proportion of eggs (transformed into probits) by corresponding fruit temperature. The grey line describes the equation: Probit oviposition cumulated = −0.79329 + 0.2602308 × *temperaure* °*C*; r² = 0.996; d.f. = 1, 200; F = 46650.75; p <0.0001; Probit standard error = 0.027; Temperature standard error = 0.001. The broken lines represent the 95% fiducial limits (r^2^ = 0.996, n = 202, p < 0.0001).

The equation obtained after probit-analysis calculated from cumulative oviposition ratios over both temperature gradients allowed the calculation of temperature ranges for oviposition activity (Fig 4). We suggest considering the temperature range for 25 to 75% cumulative oviposition as the preferred temperature, because 25% oviposition occurred at lower or higher temperatures each (Table 3).

**Table 3:** Fruit temperature preference for oviposition as given by probits, respective cumulated proportion of eggs and corresponding fruit temperature. (Calculated from equation given in legend Fig 4).

**Probit (y)**	3.36	4.16	4.33	5.0	5.67	5.84	6.64
**cumulated proportion of eggs (x)**	5%	20%	25%	50%	75%	80%	95%
**Temperature (°C)**	15.96	19.03	19.69	22.26	24.84	25.49	28.56

50% of eggs were deposited on blueberries of a temperature ranging between 19.69 to 24.84°C, and 90% oviposition occurred on fruits of a temperature range of 15.96 to 28.56°C, as calculated using the equation for linear correlation of cumulative oviposition probits with fruit temperature (Table 3).

In both temperature gradients, the lowest and highest temperatures allowing oviposition was 12.0 and 35.2°C, respectively.

During all experiments, *D*. *suzukii* dispersed in the entire experimental temperature gradient arena and did not show any abnormal behavior. Most oviposition took place where the fruit skin of blueberries came into contact with the temperature gradient bar.

## Discussion

Insects, particularly small insects such as *Drosophila* sp., are susceptible to rapid changes in body temperature, which mainly depends on ambient temperature. Lacking effective processes to adjust convenient body temperature, a fast detection of environment and body temperature is crucial for small insect survival [25]. However, a rapid detection of suboptimal or extreme temperatures should elicit an even more rapid behavioral response to move towards convenient or optimal temperatures protecting the insects from temperature damage or even death [26]. In the field flies would escape sub-optimal temperatures simply by flying away, which is not possible in our experimental set-up, where *D*. *suzukii* were only able to reach optimal temperature regions by walking on the surface of the temperature gradient bar. Despite this restriction of movements, our experimental set-up provided more accurate results because a greater distance between the roof and the temperature gradient bar would not guarantee an even temperature profile, as the air masses would mix. Flies then would continuously move around and proper assessment of fly numbers per temperature would have been impossible. If not affected by extreme temperatures, the walking behaviour and patterns were similar to that in the rearing boxes. We observed that the final choice of preferred temperature by *D*. *suzukii* was made approx. after 25 min, and fly dispersion on the temperature bar remained almost constant after that time until the end of the experiments in each individual replicate. Thus, we determined a temperature detection—response time of max. 25 min.

Previous acclimation of flies determined cold or heat tolerance as expressed by heat dependent mortality and temperature preference. Temperature preference, as a trend, positively correlated with adaptation temperature. Also, heat-dependent mortality was higher in flies adapted to 10°C than in flies adapted to 20 or 30°C.

Extrapolating our laboratory tests with summer morphs into the field, a summer generation of *D*. *suzukii* should be less susceptible to hot temperatures than spring generations leaving the hibernating sites. Sudden temperature changes, which may exceed 30°C in spring, could therefore cause high mortality in early *D*. *suzukii* generations. These findings could provide important additional information and data for modeling population dynamics of *D*. *suzukii*. Thus, the population structure in spring and summer could be better understood and calculated by models such as a preliminary mechanistic physiologically-based demographic model or a population dynamic model [27, 28]. These models can help when considering a long-term strategy for controlling this species [28]. Furthermore, we hypothesize that acclimation to lower temperatures and the resulting lower temperature preferences, perhaps in conjunction with a higher physical activity compared to flies without low temperature acclimation allows the flies to disperse better in spring to search for oviposition sites. This supports the assumptions by Tonina et al. [29], Shearer et al. [30] and Jakobs et al. [31] who reported an increased survival at 1°C, a reproductive diapause, and a temperature difference of 0.5°C between summer and winter morphs to enter the “chill coma” as well as a faster recovering at its end.

In comparison with data on *D*. *suzukii* from Kanzawa [16] and Kimura [32] and on tephritids [33], the upper and lower thresholds are equal to our studies and are close to the developmental extremes for *D*. *suzukii* [10].

It seems that the highest oviposition activity rate found in our study was at a fruit temperature of 22.26°C. The highest net reproductive rate and intrinsic rate of population increase at 22°C as described by Tochen et al. (2014) represents well the temperature optimum of reproduction biology of *D*. *suzukii*. The lower development threshold of 11.1°C [29] can also be approximately confirmed by our studies. Temperature determines the speed of preimaginal development and mortality. Thus, we hypothesize that, as a kind of brood-care, *D*. *suzukii* -females expose their eggs to temperatures optimal for their development and reduction of detrimental effects by an accurate choice of oviposition sites [28]. If so, eggs on fruits directly exposed to sun irradiation should be found rarely in summer. On the other hand, in the late evening fruits have cooled down to preferable temperatures [34], which supports the assumption of Dillon et al. (2009) who reported *D*. *suzukii* -females laying the significant higher proportion (50.9%) of their daily egg production in the period from 20:00–24:00 pm compared to the other 4 h-sections.

Transferring the results of our study to practice we hypothesize, that our findings may support recommended procedures to reduce *D*. *suzukii* infestation pressure in European viticulture. Defoliation of grapevines resulted in a 9–10°C higher temperature of Cabernet Sauvignon-berries at noon compared to shaded berries [35]. The surface temperature of exposed berries rose to > 37°C, exceeding the upper temperature threshold for oviposition activity and larval development. In blackberries *D*. *suzukii* infestation was higher in the shaded inner portion of the canopy compared to more sun-exposed locations [36].

Although we did not assess the internal fruit temperature, which is responsible for egg survival, we consider the surface temperature of superior importance for two reasons: (i) since there is no report on temperature sensing by sensilla located on or adjacent to the ovipositor of *Drosophila* species, we must assume that *D*. *suzukii* can perceive surface temperatures only, and (ii) the eggs of *D*. *suzukii* are deposited beneath the fruit surface [6] where the internal temperatures should at least be similar to the surface temperatures. On the other hand, flesh temperature of different fruits was always above air temperature either in shaded or sun-exposed fruits [23], and surface temperature has an even higher importance in oviposition behavior to avoid pernicious effects on the offspring.

Knowing the temperature adaptation capacity, thresholds and key temperature effects on oviposition of *D*. *suzukii* allows a better prediction of *D*. *suzukii* infestation pressure under different micro environmental conditions, particularly of early ripening fruit varieties.
